# Mortality prediction in pediatric postcardiotomy veno-arterial extracorporeal membrane oxygenation: A comparison of scoring systems

**DOI:** 10.3389/fmed.2022.967872

**Published:** 2022-08-04

**Authors:** Yu Jin, Peng Gao, Peiyao Zhang, Liting Bai, Yixuan Li, Wenting Wang, Zhengyi Feng, Xu Wang, Jinping Liu

**Affiliations:** ^1^Department of Cardiopulmonary Bypass, Fuwai Hospital, National Center for Cardiovascular Diseases, Chinese Academy of Medical Sciences and Peking Union Medical College, Beijing, China; ^2^Department of Pediatric Intensive Care Unit, Fuwai Hospital, National Center for Cardiovascular Diseases, Chinese Academy of Medical Sciences and Peking Union Medical College, Beijing, China

**Keywords:** veno-arterial extracorporeal membrane oxygenation, pediatric, risk prediction, in-hospital mortality, postcardiotomy

## Abstract

**Background:**

Pediatric postcardiotomy veno-arterial extracorporeal membrane oxygenation (VA-ECMO) patients have high mortality and morbidity. There are currently three scoring systems available to predict mortality: the Pediatric Extracorporeal Membrane Oxygenation Prediction (PEP) model, Precannulation Pediatric Survival After VA-ECMO (Pedi-SAVE) score, and Postcannulation Pedi-SAVE score. These methods provide risk stratification scores for pediatric patients requiring ECMO for cardiac support. However, comparative validation of these scoring systems remains scarce. We aim to assess the ability of these models to predict outcomes in a cohort of pediatric patients undergoing VA-ECMO after cardiac surgery, and identify predictors of in-hospital mortality.

**Methods:**

A retrospective analysis of 101 children admitted to Fuwai Hospital who received VA-ECMO from January 1, 2010 to December 31, 2020 was performed. Patients were divided into two groups, survivors (*n* = 49) and non-survivors (*n* = 52) according to in-hospital mortality. PEP model and Pedi-SAVE scores were calculated. The primary outcomes were the risk factors of in-hospital mortality, and the ability of the PEP model, Precannulation Pedi-SAVE and Postcannulation Pedi-SAVE scores to predict in-hospital mortality.

**Results:**

Postcannulation Pedi-SAVE score accessing the entire ECMO process had the greatest area under receiver operator curve (AUROC), 0.816 [95% confidence interval (CI): 0.733–0.899]. Pre-ECMO PEP model could predict in-hospital mortality [AUROC = 0.691 (95% CI: 0.565–0.817)], and Precannulation Pedi-SAVE score had the poorest prediction [AUROC = 0.582(95% CI: 0.471–0.694)]. Lactate value at ECMO implantation [OR = 1.199 (1.064–1.351), *P* = 0.003] and infectious complications [OR = 5.169 (1.652–16.172), *P* = 0.005] were independent risk factors for in-hospital mortality.

**Conclusion:**

Pediatric cardiac ECMO scoring systems, including multiple risk factors before and during ECMO, were found to be useful in this cohort. Both the pre-ECMO PEP model and the Postcannulation Pedi-SAVE score were found to have high predictive value for in-hospital mortality in pediatric postcardiotomy VA-ECMO.

## Introduction

Postcardiotomy veno-arterial extracorporeal membrane oxygenation (VA-ECMO) is performed ~6% of neonates and children who undergo congenital heart disease (CHD) surgery as a rescue for intractable cardiopulmonary failure (1). Despite advances in technology and experience, pediatric ECMO mortality and costs remain high (2, 3). According to the 2022 Extracorporeal Life Support Organization (ELSO) international registry, in-hospital mortality in patients with cardiac ECMO support is 56% in neonates and 46% in children (4). Diverse risk factors before and during ECMO, including patient-related variables and clinical management, are linked with significant adverse effects on clinical outcomes (2). However, the lack of well-documented prognostic prediction models and randomized controlled trials complicate the prediction of successful surgical outcomes and mortality when pediatric postcardiotomy VA-ECMO is used (5).

Development, study, and application of prediction models for ECMO has been ongoing, and prediction scores for adult respiratory ECMO (6–8), adult cardiac ECMO (9–11), pediatric respiratory and neonatal respiratory ECMO (12–15) have been well-established and validated by multiple centers. Effective predictive models play an essential role in assessing risk and predicting prognosis. Unfortunately, children with CHD have highly heterogeneous anatomies and pathophysiologies, making it challenging to develop risk prediction models for pediatric VA-ECMO patients after CHD surgery. Scoring systems for pediatric cardiac ECMO are few and have only recently appeared.

The first available prognostic model for pediatric patients who receive extracorporeal cardiopulmonary resuscitation (ECPR) or require cardiac ECMO, the Pediatric Extracorporeal Membrane Oxygenation Prediction (PEP) model (5), was published in 2018. Only recently, in 2022, were the Precannulation Pediatric Survival After VA-ECMO (Pedi-SAVE) and Postcannulation Pedi-SAVE scoring methods developed, based solely on variables stored in the ELSO registry (16). The PEP model was developed using prospectively collected data in the Bleeding and Thrombosis on ECMO (BATE), a study of eight Collaborative Pediatric Critical Care Research Network (CPCCRN) sites (5, 17). It is a pre-ECMO evaluation model which includes eight predictor variables (indication for ECMO, age, congenital diaphragmatic hernia (CDH), meconium aspiration syndrome (MAS), baseline pH in arterial blood, partial thromboplastin time, international normalized ratio (INR), and documented blood stream infection (D-BSI) prior to ECMO), and is calculated as described in: https://www.cpccrn.org/calculators/ecmoprediction/. This prognostic model was developed using the 514 ECMO runs, and externally validated by 4,342 ELSO patients (18). Mortality prediction ranges from 0 to 100 percent, with a lower score predicting better survival. The two Pedi-SAVE scoring methods were developed and validated from the data of 10,091 pediatric cardiac patients in the ELSO registry who had been supported with initial VA-ECMO. The Precannulation Pedi-SAVE score ranges from 0 to 81 points, in 5 risk categories, with a higher score predicting better survival. Eight precannulation variables (clinical group, age, race, Society of Thoracic Surgeons-European Association for Cardio-Thoracic Surgery (STAT) mortality category, pre-ECMO blood gas pH, precannulation acid buffer, total number of cardiac procedures, and indication for failure to wean from CPB) provide an effective tool for benchmarking pediatric VA-ECMO populations before ECMO initiation. Five pre-ECMO variables (clinical group, age, race, maximum STAT mortality category, pre-ECMO blood gas pH), as well as pump flow at 24 h and complications, constitute the Postcannulation Pedi-SAVE score. It has the best performance when used to evaluate the ELSO registry data, with a C-statistics probability of 0.70, compared to 0.64 in the PEP model, and 0.62 in the Precannulation Pedi-SAVE score. The Postcannulation Pedi-SAVE score is divided into 5 risk groups, with scores varying from 0 to 159. A higher mortality is predicted by a lower score. The details of these three prediction scores are shown in Table 1.

**Table 1:** Details of the pediatric ECMO prediction scores.

**Variables**		**PEP model**	**Precannulation Pedi-SAVE score**	**Postcannulation Pedi-SAVE score**
Data set		CPCCRN-BATE study	ELSO registry	
Cases		514 (<19 years)	Model development (*n* = 6,727); Validation (*n* = 3,364) (0 days−18 years)	
Study year		December 2012 to September 2014	January 2001 to December 2015	
Pre-ECMO variables	Demographics	Age	Age, Race	
	ECMO modes	VV-ECMO, VA-ECMO	VA-ECMO	
	Diagnosis	CDH, MAS, others	SVCHD, BVCHD, Primary CM, Secondary CM, pulmonary hypertension	
	Cardiac surgery	——	Maximum STAT mortality category, Total number of cardiac procedures <2, Failure to wean from CPB	Maximum STAT mortality category
	Laboratory parameters	pH, APTT, INR	pH	pH
	Special issues	Pre-ECMO documented blood infection	Precannulation acid buffer	——
Mid-ECMO variables	Pump flow (mL/kg/min)	——	——	Post-ECMO pump flow at 24 h
	Complications	——	——	Cardiovascular, Hemorrhagic, Infectious, Mechanical, Neurologic, Pulmonary, Renal
Risk groups		Ten	Five	Five
Application		Pre-ECMO evaluation	Pre-ECMO evaluation	Overall ECMO evaluation

ECMO, Extracorporeal Membrane Oxygenation; PEP, Pediatric Extracorporeal Membrane Oxygenation Prediction; Pedi-SAVE, Pediatric Survival After Veno-arterial ECMO; CPCCRN, Collaborative Pediatric Critical Care Research Network; BATE, Bleeding and Thrombosis on ECMO, ELSO, Extracorporeal Life Support Organization; VV, venovenous; VA, venoarterial; CDH, congenital diaphragmatic hernia; MAS, meconium aspiration syndrome; SVCHD, single ventricle congenital heart disease; BVCHD, biventricular congenital heart disease; CM, cardiomyopathy; STAT, Society of Thoracic Surgeons-European Association of Cardiothoracic Surgery; CPB, cardiopulmonary bypass; APTT, activated partial thromboplastin time, INR international normalized ratio.

Pediatric postcardiotomy VA-ECMO outcomes are investigated to identify factors associated with in-hospital mortality, and the utility of the three prediction models for children who received VA-ECMO support after CHD surgery are compared.

## Materials and methods

### Study population and groups

Data from 105 consecutive pediatric patients (aged younger than 18 years) who received postcardiotomy VA-ECMO from January 2010 to December 2020 at Fuwai Hospital were collected. Four patients were excluded due to ECMO running time <24 h. The remaining 101 patients were divided into two groups based on in-hospital mortality: survivors (*n* = 49), and non-survivors (*n* = 52). Demographics, pre-ECMO variables, complications, and clinical outcomes were collected. The institutional ethics board of Fuwai Hospital approved the study (NO: 2020-1346). Being a retrospective analysis, individual consent was waived.

### Outcomes and definitions

Primary outcomes were the risk factors associated with in-hospital mortality, and the value of PEP, Precannulation Pedi-SAVE, and Postcannulation Pedi-SAVE scores in predicting in-hospital mortality. Secondary outcomes included the association between the three prediction scores and clinical outcomes, and the predictive value of the PEP model and Precannulation Pedi-SAVE score for complications.

ELSO defined complications (16). Cardiovascular complications included the usage of inotropes on ECMO, cardiopulmonary resuscitation (CPR), myocardial stun, arrhythmia, hypertension requiring vasodilators, and tamponade. Hemorrhagic complications included gastrointestinal (GI) hemorrhage, cannulation or surgical site bleeding, hemolysis [plasma-free hemoglobin (pFHb) > 50 mg/dl], and disseminated intravascular coagulation (DIC). Infection was detected by blood, sputum, or urine cultures, and viral nucleic acid detection. Mechanical complications were defined as circuit changes due to circuit component clots, and oxygenator, or pump failure. Renal complications were classified as creatinine levels above 1.5 times baseline, or the need for renal replacement therapy. Neurological complications were defined as clinical symptoms (such as seizures) or neurological abnormalities revealed by imaging, such as hemorrhage, stroke, or ischemia. Pulmonary complications included pneumothorax requiring treatment, and pulmonary hemorrhage. Successful weaning from ECMO was defined as survival >48 h after weaning.

### ECMO system

Patients in our center received VA-ECMO support for the following indications: (a) Cardiac support: failure to wean from cardiopulmonary bypass (CPB); low cardiac output syndrome (LCOS); (b) ECPR; and (c) Respiratory support (19).

Patients received a right atrium-ascending aorta cannula through the original surgical incision if they were <30 kg; otherwise, a femoral vein-femoral artery cannula was performed. The ECMO system was composed of an oxygenator (Hilite 800/2400 LTTM, Medos Medizintechnik AG, Stolberg, Germany; Quadrox PLS^®^ MAQUET Cardiovascular, Hirrlingen, Germany; Sorin, Italy), a centrifugal pump (Jostra; Maquet Inc., Rastatt, Germany), and polyvinyl chloride (PVC) tubing. Priming the system was done with Plasma-Lyte A (PLA, Baxter Healthcare, Deerfield, IL, USA). Additionally, when needed, 20% human albumin, 500–1,000 units of unfractionated heparin (UFH), sodium bicarbonate, packed red blood cells (RBC), or fresh frozen plasma (FFP) were added.

### ECMO management

After ECMO initiation, pump flow was maintained at 40–220 ml/kg/min, and vasoactive drugs were gradually reduced to obtain a mean arterial pressure (MAP) of 40–70 mmHg, arterial blood oxygen saturation (SO_2_) no <95%, and venous blood SO_2_ above 70%. Ventilator parameters were set according to the lung protective principle, with positive end-expiratory pressure of 4–10 cmH_2_O, peak inspiratory pressure of <20 cmH_2_O, and a ventilation rate of 8–20 breaths/min, and fraction-inspired oxygen level of 0.3–0.6. Fentanyl and imidazole were administrated for anesthesia and sedation. An UFH dose was infused for systemic anticoagulation, and was adjusted according to activated partial thromboplastin time (APTT), activated clotting time (ACT), and chest-tube drainage. The target APTT was 50–80s, and ACT was 140–200s. Antibiotics were used prophylactically to avoid infection. The ECMO system was checked every hour for mechanical complications. Negative fluid balance was maintained with the assistance of diuretics, peritoneal dialysis, or continuous renal replacement therapy (CRRT). Routine bedside transthoracic echocardiography and chest X-rays were performed daily, while computed tomography (CT), and magnetic resonance imaging (MRI) were employed according to patient condition and the doctor's judgment. Other detailed approaches to managing pediatric postcardiotomy VA-ECMO have been previously described in literature (19).

### Statistical analyses

Continuous variables were represented by median (interquartile range; IQR), and analyzed by Mann–Whitney *U*-test. Categorical variables were expressed as a percentage of *n* (%), and analyzed by Fisher's exact test or chi-square test. Logistic stepwise regression analyses were undertaken to assess predictors of in-hospital mortality. All variables were evaluated for correlation with survival to discharge through univariate analysis. Univariable analysis factors with *P* < 0.10 were entered into the models, followed by forward stepwise multiple logistic regression analysis to identify predictors of in-hospital mortality. PEP model, Precannulation Pedi-save, and Postcannulation Pedi-SAVE scores were calculated for all patients. Area under the receiver operating characteristic curve (AUROC) and Hosmer-Lemeshow (HL) goodness-of-fit test were used to assess the performance of the three prediction scores. Spearman rank correlation was used to test for correlation among ECMO duration, hospital length of stay, ICU length of stay, ventilation time, and prediction scores. The receiver operating characteristic (ROC) curve was analyzed to explore the predictive value of the PEP model and Precannulation Pedi-SAVE score for complications. The data was analyzed and visualized with SPSS 22.0 (SPSS, Inc., Chicago, IL, USA), and GraphPad Prism 8.0 (GraphPad Software, Inc., San Diego, CA, USA), respectively.

## Results

### Demographics and pre-ECMO variables

The median age of patients at ECMO implantation in the cohort was 12.7 (6.0, 39.3) months, and the median weight was 8.5 (5.9, 12.8) kilograms. Minimum age was 3 days, and minimum weight was 2.6 kilograms. More than half (63.4%) of the ECMOs were performed for cardiac support, including 51 patients who were unable to wean off CPB, and 13 patients who had LCOS. About a quarter (24.8%) of the patients received ECPR, and ECMO for respiratory support accounted for 12 cases. Of the patients in this cohort which were diagnosed with heterogeneous CHD, transposition of the great arteries (TGA; 19.8%) was the most common diagnosis (Supplementary Table 1), while arterial switch operations (ASO; 24.8%) were the most common surgical procedure (Supplementary Table 2).

There were no significant differences in gender, weight, age, cardiac surgery history, preoperative infection, Risk Adjustment for Congenital Heart Sugery-1 (RACHS-1) class, STAT mortality category (20), CPB time, clamp time, MAP, pH, APTT, vasoactive-inotropic score (VIS) (21), or precannulation acid buffer requirement at ECMO implantation between the groups. A greater number of survivors than non-survivors received ECMO for cardiac support (*P* = 0.022). The significant difference between the groups were in lactate (*P* = 0.009), INR (*P* = 0.032) at ECMO implantation, and post-ECMO pump flow at 24 h (*P* = 0.008; Table 2).

**Table 2:** Patient characteristics of survivors and non-survivors.

**Variables**	**Total** **(*n* = 101)**	**Survivors** **(*n* = 49)**	**Non-survivors** **(*n* = 52)**	***P*-value**
**Demographics, pre-ECMO, and mid-ECMO variables**				
Male sex	63 (62.4)	30 (61.2)	33 (63.5)	0.840
Weight (kg)	8.5 (5.9, 12.8)	9.4 (5.9, 13.9)	8.3 (5.9, 12.3)	0.311
Age (m)	12.7 (6.0, 39.3)	13.9 (6.4, 42.4)	10.1 (5.4, 37.3)	0.425
RACHS-1 class	3.0 (2.0, 4.0)	3.0 (2.5, 4.0)	3.0 (2.0, 4.0)	0.529
Redo-cardiac surgery	27 (26.7)	14 (28.6)	13 (25.0)	0.822
STAT mortality category	4.0 (2.0, 4.0)	3.0 (2.0, 4.0)	4.0 (2.0, 4.0)	0.120
CPB time (min)	259.0 (156.5, 366.5)	269.0 (161.0, 379.0)	246.0 (145.3, 342.0)	0.550
Clamp time (min)	119.0 (74.5, 153.5)	117.0 (76.5, 158.0)	125.0 (73.3, 151.0)	0.921
**Indications**				
ECPR	25 (24.8)	8 (16.3)	17 (32.7)	0.068
Cardiac	64 (63.4)	37 (75.5)	27 (51.9)	0.022
Respiratory	12 (11.9)	4 (8.2)	8 (15.4)	0.360
Preoperative infection	9 (8.9)	4 (8.2)	5 (9.6)	1.000
PH at ECMO implantation	7.4 (7.3, 7.5)	7.4 (7.4, 7.5)	7.4 (7.3, 7.5)	0.260
APTT at ECMO implantation	65.3 (44.3, 95.8)	54.1 (41.7, 89.8)	67.2 (47.8, 99.6)	0.252
INR at ECMO implantation	1.4 (1.2, 1.7)	1.3 (1.2, 1.6)	1.5 (1.2, 1.9)	0.032
MAP at ECMO implantation	46.0 (39.5, 58.0)	46.0 (40.0, 58.5)	46.0 (38.3, 56.0)	0.540
Lactate at ECMO implantation	7.6 (4.8, 11.1)	6.3 (4.4, 9.0)	8.6 (5.6, 13.9)	0.009
VIS at ECMO implantation	27.0 (17.0, 45.0)	22.0 (16.0, 42.5)	28.5 (18.0, 47.8)	0.222
Precannulation acid buffer	38 (37.6)	15 (30.6)	23 (44.2)	0.218
Post-ECMO pump flow at 24 h (mL/kg/min)	93.6 (76.0, 114.9)	85.5 (69.4, 103.3)	102.4 (80.0, 122.7)	0.008
**Complications**				
Hemorrhagic	70 (69.3)	30 (61.2)	40 (76.9)	0.130
Infectious	47 (46.5)	16 (32.7)	31 (59.6)	0.009
Mechanical	20 (19.8)	6 (12.2)	14 (26.9)	0.082
Neurological	14 (13.9)	2 (4.1)	12 (23.1)	0.008
Pulmonary	7 (6.9)	1 (2.0)	6 (11.5)	0.113
Renal	74 (73.3)	28 (57.1)	46 (88.5)	0.001
**Prediction scores**				
PEP model	45.0 (40.0, 56.0)	42.0 (37.0, 50.5)	50.5 (42.0, 60.5)	0.003
Precannulation Pedi-SAVE	49.0 (46.0, 53.0)	50.0 (47.0, 53.0)	49.0 (45.3, 52.8)	0.153
Postcannulation Pedi-SAVE	97.0 (85.0, 110.3)	108.0 (98.5, 119.0)	89.0 (77.5, 97.0)	<0.001
**Clinical outcomes**				
ECMO duration (h)	123.0 (91.5, 167.0)	101.3 (89.5, 135.5)	145.5 (102.5, 211.8)	0.001
Successful Weaning	70 (69.3)	49 (100.0)	21 (40.4)	<0.001
Hospital length of stay (d)	42.0 (22.0, 63.0)	51.0 (36.0, 84.5)	50.5 (42.0, 60.5)	<0.001
ICU length of stay (d)	28.0 (11.5, 48.0)	33.0 (23.5, 56.0)	14.0 (7.0, 37.3)	<0.001
Ventilation time (h)	494.0 (203.5, 853.0)	567.0 (284.0, 967.5)	289.0 (144.8, 818.0)	0.006

Continuous data are presented as median (interquartile range) and categorical data as n (percent).

ECMO, Extracorporeal Membrane Oxygenation; RACHS-1, Risk Adjustment for Congenital Heart Sugery-1; STAT, Society of Thoracic Surgeons-European Association of Cardiothoracic Surgery; CPB, cardiopulmonary bypass; ECPR, extracorporeal cardiopulmonary resuscitation; MAP, mean arterial pressure; VIS, vasoactive-inotropic score; APTT, activated partial thromboplastin time, INR international normalized ratio; PEP, Pediatric Extracorporeal Membrane Oxygenation Prediction; Pedi-SAVE, Pediatric Survival After Veno-arterial ECMO; ICU, intensive care unit.

### Complications and clinical outcomes

The occurrence of infectious (*P* = 0.009), neurological (*P* = 0.008), and renal (*P* = 0.001) complications were positively correlated with in-hospital mortality. All patients had cardiovascular complications. Seven patients suffered pulmonary hemorrhages. Hemorrhagic, mechanical, and pulmonary complications were not significantly associated with mortality (Table 2).

Seventy patients (69.3%) were successfully weaned from ECMO. Forty-nine children survived, and the overall survival rate was 48.5%. Median ECMO duration was 123.0 (91.5, 167.0) hours, and median hospital stay was 42.0 (22.0, 63.0) days. Clinical outcomes were significantly different in ECMO duration, successful weaning rate, mechanical ventilation time, ICU length of stay, and total hospital length of stay.

### Risk factors of in-hospital mortality

After univariate logistic analysis, ECMO for cardiac support (*P* = 0.015), pre-ECMO INR (*P* = 0.028), lactate at ECMO implantation (*P* = 0.005), post-ECMO pump flow at 24 h (*P* = 0.019), hemorrhagic (*P* = 0.090), infectious (*P* = 0.007), mechanical (*P* = 0.070), neurological (*P* = 0.014), pulmonary (*P* = 0.095), and renal (*P* = 0.001) complications were all associated with in-hospital mortality. These variates were entered into multivariate analysis. In a multiple logistic regression adjusted for other factors mentioned above, lactate at ECMO implantation and infection during ECMO independently increased the odds of in-hospital mortality (Table 3).

**Table 3:** Multivariable logistic regression analysis: independent predictors of in-hospital mortality.

**Variables**	***P*-value**	**OR**	**95% CI**
Lactate at ECMO implantation	0.003	1.199	1.064–1.351
Infection during ECMO	0.005	5.169	1.652–16.172

OR, odds ratio; CI, confidence interval; ECMO, extracorporeal membrane oxygenation.

### Predictive values of PEP model, Precannulation Pedi-SAVE score, and Postcannulation Pedi-SAVE score

**Figure 1:**
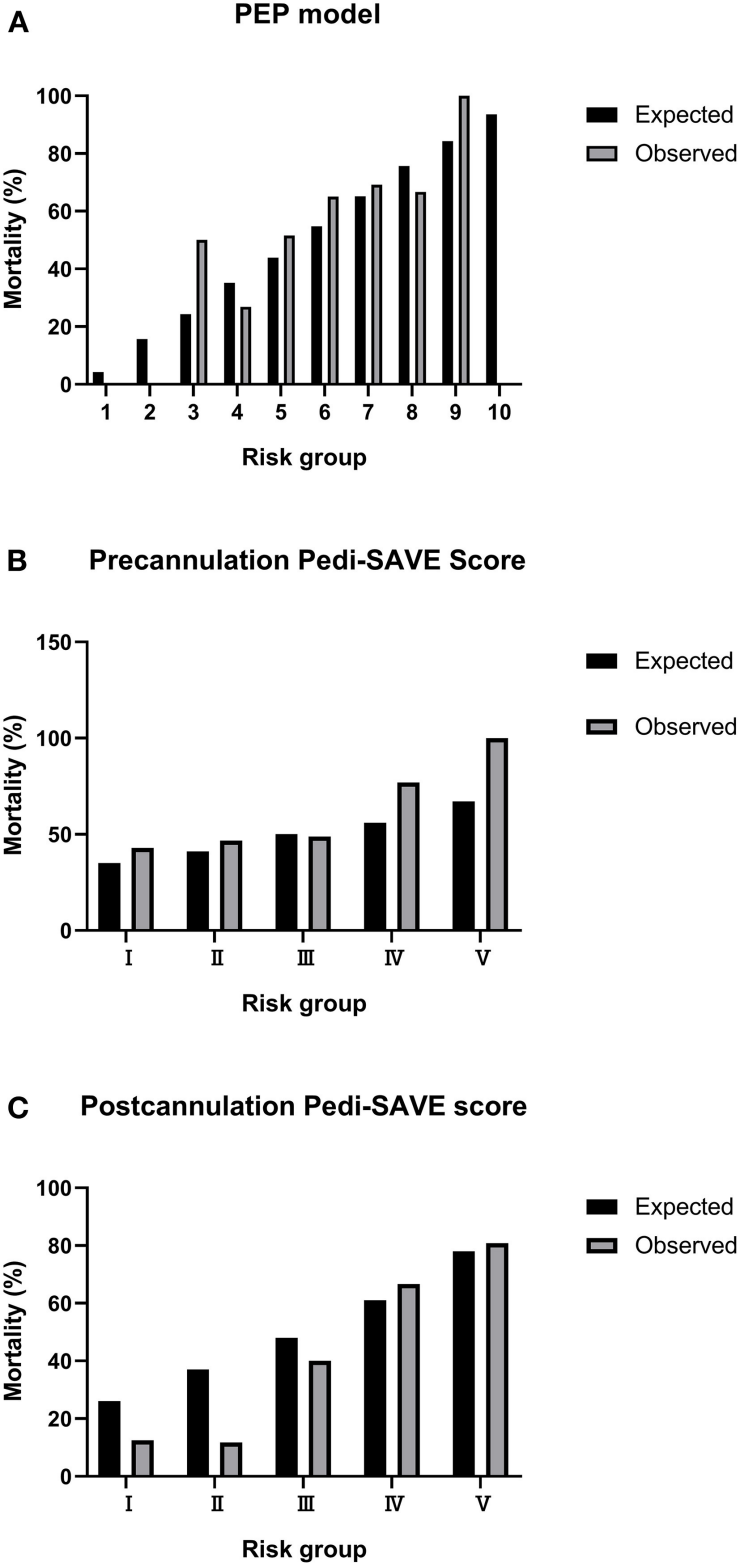
Calibration plots for observed to expected mortality. PEP, Pediatric Extracorporeal Membrane Oxygenation Prediction; Pedi-SAVE, Pediatric Survival After Veno-arterial Extracorporeal Membrane Oxygenation. **(A)** PEP model. **(B)** Precannulation Pedi-SAVE score. **(C)** Postcannulation Pedi-SAVE score.

There was a significant difference in the PEP model and Postcannulation Pedi-SAVE score between non-survivors and survivors, while the Precannulation Pedi-SAVE score was a poor predictor of death (Table 2). Observed mortality for the Precannulation Pedi-SAVE score and PEP model tested weakly paralleled expected mortality; the two scores had decreased accuracy in low-risk groups where higher than expected deaths occurred. The correlation was strongest for Postcannulation Pedi-SAVE score, where the data set had a similar distribution of predicted mortality (Figure 1).

**Figure 2:**
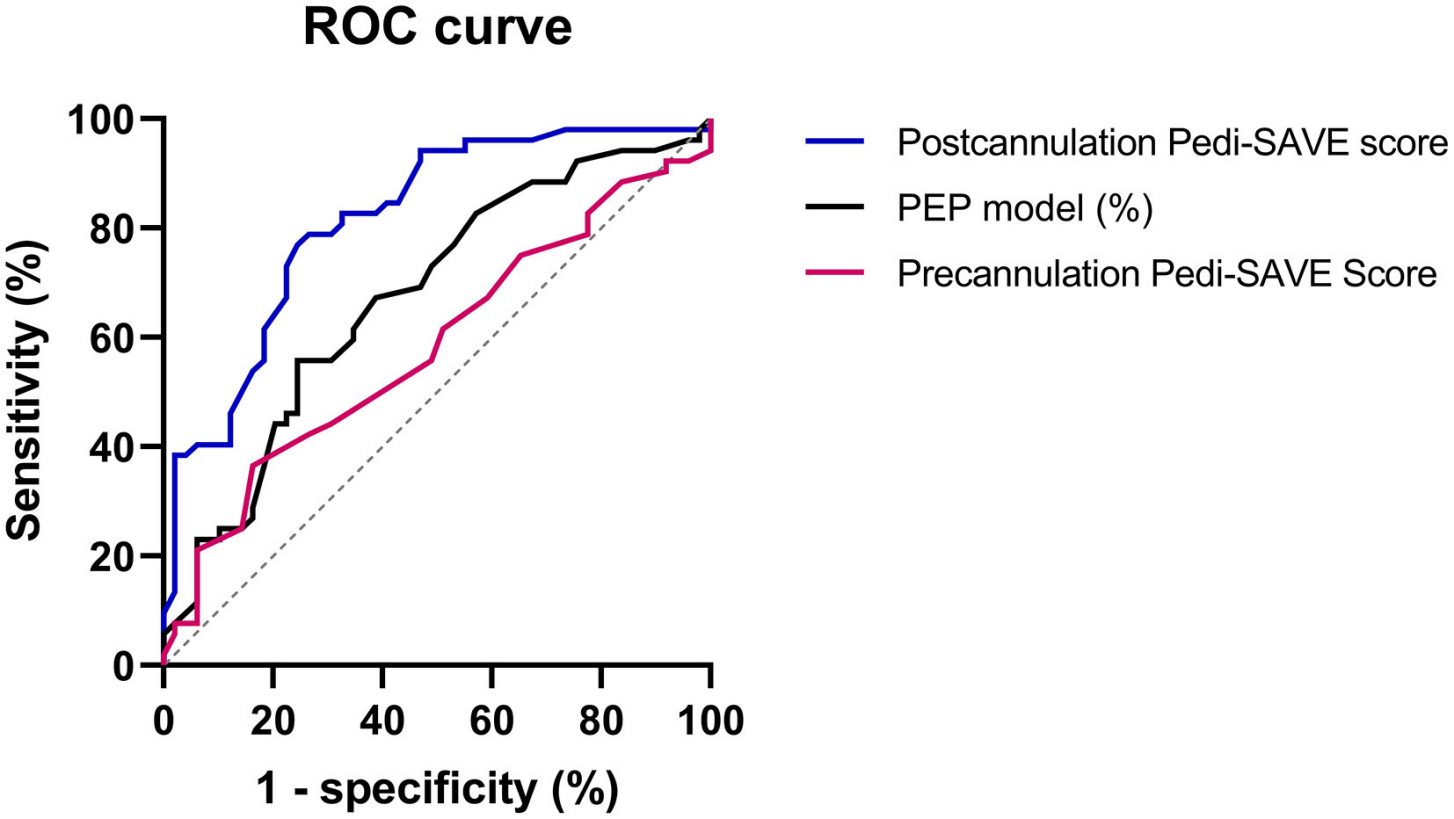
The receiver operating characteristic curve of prediction scores. ROC, receiver operating characteristic; PEP, Pediatric Extracorporeal Membrane Oxygenation Prediction; Pedi-SAVE, Pediatric Survival After Veno-arterial Extracorporeal Membrane Oxygenation; ECMO, extracorporeal membrane oxygenation.

In ROC curve analysis, the Postcannulation Pedi-SAVE score demonstrated the greatest predictive ability, with an AUROC of 0.816 (95% CI: 0.733–0.899). The PEP model also showed high discrimination for in-hospital mortality as a risk adjustment tool, with an AUROC of 0.691 (95% CI: 0.565–0.817; Table 4, Figure 2).

**Table 4:** Performance of prediction scores.

**Prediction scores**	**AUROC** **(95% CI)**	**Standard** **Error**	**HL test** ***p*-value**
**In-hospital mortality**			
PEP model	0.691 (0.565–0.817)	0.064	0.856
Precannulation Pedi-SAVE score	0.582 (0.471–0.694)	0.057	0.522
Postcannulation Pedi-SAVE score	0.816 (0.733–0.899)	0.042	0.264
**ECMO weaning failure**			
PEP model	0.694 (0.578–0.809)	0.059	0.629
Precannulation Pedi-SAVE score	0.535 (0.410–0.661)	0.064	0.821
Postcannulation Pedi-SAVE score	0.769 (0.667–0.870)	0.052	0.068

AUROC, area under the receiver operating characteristic curve; HL, Hosmer-Lemeshow; PEP, Pediatric Extracorporeal Membrane Oxygenation Prediction; Pedi-SAVE, Pediatric Survival After Veno-arterial ECMO; ECMO, extracorporeal membrane oxygenation.

We further explored the associations with clinical outcomes among the three prediction models, and the distinguishing values of the PEP model and Precannulation Pedi-SAVE score for various complications. The PEP model showed predictive values for neurological complications [AUROC = 0.676 (95% CI: 0.552–0.801)], and renal complications [AUROC = 0.659 (95% CI: 0.542–0.775)]. Additionally, the PEP model [AUROC = 0.694 (95% CI: 0.578–0.809)] and Postcannulation Pedi-SAVE score [AUROC = 0.769 (95% CI: 0.667–0.870)] demonstrated predictive ability for ECMO weaning failure (Table 4). Hospital length of stay was correlated with the PEP model (*r* = −0.293, *P* = 0.003) and the Postcannulation Pedi-SAVE score (*r* = 0.260, *P* = 0.009). ECMO duration was negatively associated with the Postcannulation Pedi-SAVE score (*r* = −0.214, *P* = 0.032). A weak relationship existed between mechanical ventilation time (*r* = −0.252, *P* = 0.011), ICU length of stay (*r* = −0.228, *P* = 0.022) and the PEP model.

As cardiac support was the most common indication in this cohort, and the two Pedi-SAVE scores established by ELSO were based on this indication, a subgroup analysis of the three predictive scores was done for the 64 patients receiving VA-ECMO for cardiac support. We found that all scores had good discrimination for in-hospital mortality, with the PEP model, Precannulation Pedi-SAVE score and Postcannulation Pedi-SAVE score having an AUROC of 0.649 (95% CI: 0.509–0.789), 0.664 (95% CI: 0.527–0.802), and 0.832 (95% CI: 0.736–0.929), respectively. To reduce selective bias, we supplemented these four patients in the study to investigate the predictive power of the three scoring models in 105 consecutive patients during the study period. Patients with no 24-h flow data scored 0 on the corresponding variable in the Postcannulation Pedi-SAVE score. Postcannulation Pedi-SAVE score demonstrated the most excellent predictive power, with an AUROC of 0.823 (95% CI: 0.743–0.903), followed by PEP model, with an AUROC of 0.682 (95% CI: 0.580–0.785). Precannulation Pedi-SAVE score performance was poor, with an AUROC of 0.586 (95 % CI: 0.477–0.696; Supplementary Table 4). In addition, the demographic and clinical variables of the 105 patients were shown in Supplementary Table 5.

## Discussion

VA-ECMO can be used as a rescue therapy for failure to wean from CPB, LCOS, cardiac arrest (CA), and acute respiratory distress syndrome (ARDS) after CHD surgery, providing time for cardiopulmonary recovery. However, it is accompanied by high morbidity and mortality; coagulopathies, renal injury, and infection are common complications of pediatric postcardiotomy VA-ECMO (17, 22, 23). Many single risk factors before and during ECMO can predict mortality after VA-ECMO, such as renal failure, lactate level, and clearance (24, 25). However, conclusions are limited to single-center experience and generalizability. Therefore, a prognostic prediction model that simultaneously considers multiple risk factors and assigns corresponding weights according to their relative importance can help us make individualized assessments.

Our study is the first cohort simultaneously validating all currently available prognostic prediction scores for pediatric VA-ECMO. Our results showed that the PEP model and Postcannulation Pedi-SAVE score were significantly associated with survival to discharge, while Precannulation Pedi-SAVE score demonstrated no difference between survivors and non-survivors. The Postcannulation Pedi-SAVE score showed the most potent discriminatory ability in ROC analysis, with an AUROC above 0.8. In this cohort, 48.5% of patients survived to discharge. Lactate at ECMO implantation and infectious complications were independent risk factors for in-hospital mortality.

The PEP model is the first mortality prediction score that can be applied to all pediatric ECMO patients without excluding age or ECMO indication (5). Using our data set from all VA-ECMO subjects, we found that the AUROC of the PEP model for predicting in-hospital mortality was 0.691, lower than the original study's 0.75. Compared with the BATE study (17), our study's population had a lower proportion of neonates (5.0 vs. 51.9%), and a higher proportion of infants (44.6 vs. 23.7%) and children (47.5 vs. 15.6%). More patients received ECMO for cardiac support (63.4 vs. 40.3%) and ECPR (24.8 vs. 13.6%). There were no CDH and MAS; all children were diagnosed with CHD (100 vs. 37.9%). As for laboratory parameters, our subjects had a higher APTT (65.3 vs. 43.7s), while INR (1.4 vs. 1.5), and pH in arterial blood (7.4 vs. 7.3) were similar. Our subjects had no D-BSI prior to ECMO (0 vs. 5.3%), and 8.9% of patients had preoperative respiratory infections. Median ECMO duration (123.0 vs. 120.0 h) was similar for both cohorts. In-hospital mortality was 44.9% in the original study, and 51.5% in our study. The PEP model has eight variables, while our subject data only included 5 parameters: age, indication, pH, APTT, and INR. This may be part of the reason for slightly different results in our center compared to the original study. As all patients required VA-ECMO after cardiac surgery, those who could not be weaned from CPB account for 50%, and coagulopathies are widespread in our subjects. The incidence of hemorrhagic complications is as high as 69.3%. APTT and INR can be used to assess the coagulation status of patients at ECMO implantation. The PEP model was associated with RBC transfusion (mL/kg/d; *r* = 0.204, *P* = 0.047) in our subjects. Thus, this model had a high discrimination ability for in-hospital mortality in our study. Moreover, it was also negatively correlated with recovery indexes: total hospital length of stay, ICU length of stay, and mechanical ventilation time.

Pedi-SAVE scores are tools for risk adjustment and benchmarking in pediatric cardiac patients supported with VA-ECMO to predict survival (16). The C-statistics of the Precannulation Pedi-SAVE score and Postcannulation Pedi-SAVE score in the 6,727 patient development dataset, and 3,364 patient internal validation dataset were 0.62 and 0.64, 0.70 and 0.71, respectively. In our subjects, the discriminative ability of the Precannulation Pedi-SAVE score was significantly lower (AUROC: 0.582), while the Postcannulation Pedi-SAVE score had highly predictive values (AUROC: 0.816). Compared to the ELSO registry, our cohort was older, and included a higher percentage of infants (44.6 vs. 29.0%) and pediatric patients (50.5 vs. 25.2%). Biventricular congenital heart disease (BVCHD; 92.0 vs. 45.4%) was the predominant diagnosis, and there were few patients with single ventricle congenital heart disease (SVCHD; 8.0 vs. 27.8%). All patients in our center were postcardiotomy (100 vs. 40.0%), a larger number of children were in the high-grade STAT mortality category (above 3; 50.5 vs. 35.2%), and more patients required precannulation acid buffer (37.6 vs. 23.9%). Regarding indications, failure to wean from CPB (50.5 vs. 38.5%) and respiratory support percentages (11.9 vs. 3.7%) were higher than in the original study, while LCOS was lower (12.9 vs. 68.2%). After ECMO initiation, a lower median 24-h pump flow (94 vs. 112 mL/kg/min) was found in our study population. Complications, including cardiovascular (100 vs. 68.3%), hemorrhagic (69.3 vs. 46.7%), renal (73.3 vs. 39.5%), and infection (46.5 vs. 9.2%) were much higher than the ELSO multicenter dataset, while mechanical complications (19.8 vs. 34.9%) were lower. Neurologic (excluding brain death; 13.9 vs. 15.9%) and pulmonary (6.9 vs. 7.7%) complications were comparable in both cohorts. Median ECMO duration (123 vs. 113 h) and survival to discharge (48.5 vs. 49.5%) were also not significantly different from the original study. Among the eight pre-ECMO risk factors in the Precannulation Pedi-SAVE score, 92.0% of the patients had BVCHD, 73.3% received primary cardiac surgery, and 70.2% had pre-ECMO pH levels between 7.3 and 7.5. These factors cause the Precannulation Pedi-SAVE score to center around 45–55, and make it difficult to distinguish risk levels effectively. The Postcannulation Pedi-SAVE score included complications that significantly impacted clinical outcomes, and each complication had a different weight. Lack of neurologic, pulmonary, renal, or infectious complications distinctly benefit survival. Our subjects were evenly distributed across the five risk groups of the Postcannulation Pedi-SAVE score, and observed mortality was highly parallel to predicted mortality. Notably, in the cardiac support subgroup excluding ECPR, both Pedi-SAVE scores had high predictive values for in-hospital mortality. Patients undergoing ECPR have varying degrees of persistent hypoperfusion and ischemia-hypoxic injury, so there may be hyperlactatemia and organ damage at ECMO implantation (26). However, the Precannulation Pedi-SAVE score does not include these corresponding risk factors, and has limited application in this population.

As a marker of tissue perfusion, the lactate value can reflect the balance of oxygen demand and supply in macro- and micro-circulation (27). Our study found that lactate at ECMO implantation was predictive of in-hospital mortality, with an AUROC of 0.650 (95% CI 0.543–0.757). The cut-off value was 7 mmol/L (sensitivity 65.4%, specificity 61.2%; *P* = 0.009). This predictor is easily measured for pre-ECMO risk evaluation and may improve patient selection. Hyperlactatemia indicates decompensated oxygen metabolism, which leads to tissue and organ damage. Fux et al. found that arterial lactate level before VA-ECMO initiation was an independent risk factor of 90-day mortality in postcardiotomy cardiogenic shock patients (27). Moreover, an earlier study in our center pointed out that pre-ECMO lactate was a predictor of acute renal failure during pediatric postcardiotomy ECMO (28).

We observed a higher infection prevalence (46.5%) than in other reports (12–42%) (29–31). More than half of the patients in our center who received postcardiotomy VA-ECMO had delayed chest closure, which increases the risk of infection (29). Positive bacterial cultures of respiratory secretions were the most common type of infection, occurring in 20.8% of our cohort (Supplementary Table 3). The most common pathogen was Gram-negative bacilli, which is associated with ventilator-associated pneumonia (VAP) (32). Nosocomial infections are associated with worse outcomes, particularly with Gram-negative bacteria infection (30, 32, 33). Our findings demonstrate that infectious complications lead to a 5-fold increase in risk of mortality. The epidemiology of infection during ECMO varies widely, and the diagnosis of nosocomial infection remains challenging, with a lack of evidence supporting biomarkers such as procalcitonin and C-reactive protein (34). Although antibiotic prophylaxis is used in half to three-quarters of ECMO centers, its effectiveness needs to be confirmed by research (35–37). The high incidence of VAP in our center suggests that prophylactic antibiotics may not be suitable for all patients.

Limitations of our study include the small number of subjects, and the limited extrapolation value of the results due to variation in clinical practice across centers. A retrospective study analyzing incomplete data may cause potential inaccuracies in the data and potential selection bias.

## Conclusion

Pre-ECMO lactate level and mid-ECMO infectious complications significantly increase the odds of in-hospital mortality. The pediatric cardiac ECMO scoring system, including multiple risk factors before and during ECMO, are helpful in our population. The pre-ECMO PEP model and the whole-course Postcannulation Pedi-SAVE score have a high predictive value for in-hospital mortality in pediatric postcardiotomy VA-ECMO. Given the heterogeneity of CHD, prediction scores should not replace individual assessment and prognostication. Further analysis of the risk factors associated with adverse outcomes on pediatric VA-ECMO support is required in the future to create more accurate risk prediction models.

## Data availability statement

The raw data supporting the conclusions of this article will be made available by the authors, without undue reservation.

## Ethics statement

The studies involving human participants were reviewed and approved by the Institutional Ethics Board of Fuwai Hospital. Written informed consent from the participants' legal guardian/next of kin was not required to participate in this study in accordance with the national legislation and the institutional requirements.

## Author contributions

JL and YJ: conception and design. LB, PZ, YL, PG, and WW: administrative support. XW, JL, and ZF: provision of study material or patients. YJ: collection and assembly of data, data analysis, and interpretation. All authors contributed to the article, manuscript writing and approved the submitted version.

## Funding

This work was supported by CAMS Innovation Fund for Medical Sciences (2020-I2M-C&T-B-063) and Fundamental Research Funds for the Central Universities (NO. 3332021022).

## Conflict of interest

The authors declare that the research was conducted in the absence of any commercial or financial relationships that could be construed as a potential conflict of interest.

## Publisher's note

All claims expressed in this article are solely those of the authors and do not necessarily represent those of their affiliated organizations, or those of the publisher, the editors and the reviewers. Any product that may be evaluated in this article, or claim that may be made by its manufacturer, is not guaranteed or endorsed by the publisher.
