# Supplementary material for: Mortality prediction in pediatric postcardiotomy veno-arterial extracorporeal membrane oxygenation: A comparison of scoring systems
Source: Front Med (Lausanne). 2022 Aug 4;9:967872. doi: 10.3389/fmed.2022.967872 (PMC9386139; doi:10.3389/fmed.2022.967872)
Supplement: Supplementary file 1 [file Data_Sheet_1.pdf]

Supplementary Table 1. Main diagnosis

Diagnosis	All (n=101)	Survivors (n=49)	Non-survivors (n=52)
**TGA**	20 (19.8)	10 (20.4)	10 (19.2)
**DORV**	14 (13.9)	4 (8.2)	10 (19.2)
**TOF**	16 (15.8)	7 (14.3)	9 (17.3)
**RVOTO**	6 (5.9)	5 (10.2)	1 (1.9)
**VSD**	7 (6.9)	3 (6.1)	4 (7.7)
**PA**	8 (7.9)	4 (8.2)	4 (7.7)
**SV**	6 (5.9)	2 (4.1)	4 (7.7)
**TECD**	4 (4.0)	2 (4.1)	2 (3.8)
**Supra-aortic stenosis**	3 (3.0)	2 (4.1)	1 (1.9)
**ALCAPA**	6 (5.9)	5 (10.2)	1 (1.9)
**Ebstein's anomaly**	3 (3.0)	2 (4.1)	1 (1.9)
**CoA**	2 (2.0)	2 (4.1)	0 (0.0)
**HRHS**	2 (2.0)	0 (0.0)	2 (3.8)
**AS+PS**	1 (1.0)	1 (2.0)	0 (0.0)
**ASD + atrial mass**	1 (1.0)	0 (0.0)	1 (1.9)
**AA**	1 (1.0)	0 (0.0)	1 (1.9)
**HLHS**	1 (1.0)	0 (0.0)	1 (2.0)

Notes: categorical data are presented as n (percent). TGA, transposition of great arteries; DORV, double outlet right ventricle; TOF, tetralogy of Fallot; ROVTO, right ventricular outflow tract obstruction; VSD, ventricle septal defect; PA, pulmonary atresia; SV, single ventricle; TECD, total endocardial cushion defect; ALCAPA, anomalous origin of left coronary artery from the pulmonary artery; CoA, coarctation of aorta; HRHS, Hypoplastic right heart syndrome; AS, aortic stenosis; PS, pulmonary stenosis; ASD, atrial septal defect; AA, aortic atresia; HLHS, hypoplastic left heart syndrome.

Supplementary Table 2. Primary Procedure

Procedure	All (n=101)	Survivors (n=49)	Non-survivors (n=52)
**Arterial switch operation**	25 (24.8)	9 (18.4)	16 (30.8)
**Rastelli procedure**	6 (5.9)	3 (6.1)	3 (5.8)
**DRT**	4 (4.0)	1 (2.0)	3 (5.8)
**TOF complete surgical repair**	16 (15.8)	7 (14.3)	9 (17.3)
**VOTO repair**	6 (5.9)	5 (10.2)	1 (1.9)
**VSD closure**	7 (6.9)	3 (6.1)	4 (7.7)
**PA complete surgical repair**	5 (5.0)	4 (8.2)	1 (1.9)
**Modified Blalock-Taussig shunt**	1 (1.0)	0 (0.0)	1 (1.9)
**Sano shunt**	1 (1.0)	1 (2.0)	0 (0.0)
**Fontan procedure**	5 (5.0)	2 (4.1)	3 (5.8)
**TECD repair**	4 (4.0)	2 (4.1)	2 (3.8)
**Supra-aortic stenosis repair**	3 (3.0)	2 (4.1)	1 (1.9)
**ALCAPA repair**	6 (5.9)	5 (10.2)	1 (1.9)
**Ebstein's anomaly repair**	3 (3.0)	2 (4.1)	1 (1.9)

**Glenn procedure**	3 (3.0)	0 (0.0)	3 (5.8)
**CoA repair**	2 (2.0)	2 (4.1)	0 (0.0)
**Bentall +Konno procedure**	1 (1.0)	1 (2.0)	0 (0.0)
**ASD closure+atrial mass resection**	1 (1.0)	0 (0.0)	1 (1.9)
**Ascending aortoplasty**	1 (1.0)	0 (0.0)	1 (1.9)
**Norwood I procedure**	1 (1.0)	0 (0.0)	1 (1.9)

Notes: categorical data are presented as n (percent). DRT, double root translocation; TOF, tetralogy of Fallot; OVTO, ventricular outflow tract obstruction; VSD, ventricle septal defect; PA, pulmonary atresia; TECD, total endocardial cushion defect; ALCAPA, anomalous origin of left coronary artery from the pulmonary artery; CoA, coarctation of aorta; ASD, atrial septal defect.

Supplementary Table 3. Infectious complications

Infectious complications (n=47)	Overall (n=101)
**Pathogen type**	
**Bacterial**	27 (26.7)
**Fungal**	11 (10.9)
**Viral**	6 (5.9)
**Multiple**	3 (3.0)
**Site of infection**	
**Respiratory**	35 (34.7)
**Blood**	6 (5.9)
**Urine**	2 (2.0)
**Multiple**	4 (4.0)

Supplementary Table 4. Performance of prediction scores

Prediction Scores	AUROC (95% CI)	Standard Error	HL test p value
**In-hospital mortality**			
PEP model	0.682 (0.580-0.785)	0.052	0.865
Precannulation Pedi-SAVE score	0.586 (0.477-0.696)	0.056	0.334
Postcannulation Pedi-SAVE score	0.823 (0.743-0.903)	0.041	0.532

Notes: AUROC, area under the receiver operating characteristic curve; HL, Hosmer-Lemeshow; PEP, Pediatric Extracorporeal Membrane Oxygenation Prediction; Pedi-SAVE, Pediatric Survival After Veno-arterial ECMO; ECMO, extracorporeal membrane oxygenation.

Supplementary Table 5. Patients' characteristics of Survivors and Non-survivors

Variables	Total (n=105)	Survivors (n=51)	Non-survivors (n=54)	P value
**Demographics, pre-ECMO and mid-ECMO variables**				
Male sex	65 (61.9)	31 (60.8)	34 (63.0)	0.843
Weight (kg)	8.5 (5.8, 13.0)	9.7 (6.0, 14.0)	7.9 (5.7, 11.8)	0.116

Age (m)	12.7 (5.5, 40.6)	15.6 (6.5, 43.1)	9.7 (4.8, 35.4)	0.190
Redo-cardiac surgery	27 (25.7)	14 (27.5)	13 (24.1)	0.824
RACHS-1 class	3.0 (2.0, 4.0)	3.0 (2.0, 4.0)	3.0 (2.0, 4.0)	0.389
STAT mortality category	4.0 (2.0, 4.0)	3.0 (2.0, 4.0)	4.0 (2.0, 4.0)	0.084
CPB time (min)	259.0 (161.0, 376.5)	267.0 (162.0, 374.0)	256.0 (149.8, 388.0)	0.842
Clamp time (min)	119.0 (74.5, 153.5)	117.0 (76.5, 158.0)	125.0 (73.3, 151.0)	0.921
Indications				
ECPR	25 (23.8)	8 (15.7)	17 (31.5)	0.069
Cardiac	68 (64.8)	39 (76.5)	29 (53.7)	0.024
Respiratory	12 (11.4)	4 (7.8)	8 (14.8)	0.361
Preoperative infection	10 (9.5)	4 (7.8)	6 (11.1)	0.742
PH at ECMO implantation	7.4 (7.3, 7.5)	7.4 (7.4, 7.5)	7.4 (7.3, 7.5)	0.151
APTT at ECMO implantation	67.2 (44.8, 103.0)	66.9 (44.6, 109.7)	67.9 (44.9, 100.8)	0.972
INR at ECMO implantation	1.4 (1.2, 1.7)	1.3 (1.2, 1.5)	1.5 (1.2, 1.9)	0.049
MAP at ECMO implantation	46.0 (39.5, 58.0)	47.0 (40.0, 60.0)	45.5 (38.8, 56.0)	0.326
Lactate at ECMO implantation	7.6 (4.9, 11.2)	6.4 (4.4, 9.0)	8.9 (5.7, 14.1)	0.004
VIS at ECMO implantation	27.0 (17.0, 45.5)	22.0 (16.0, 43.0)	28.5 (17.5, 48.5)	0.282
Precannulation acid buffer	38 (36.2)	15 (29.4)	23 (42.6)	0.223
Post-ECMO pump flow at 24 hours* (mL/kg/min)	93.6 (76.0, 114.9)	85.5 (69.4, 103.3)	102.4 (80.0, 122.7)	0.008
**Complications**				
Hemorrhagic	72 (68.6)	31 (60.8)	41 (75.9)	0.140
Infectious	47 (44.8)	16 (31.4)	31 (57.4)	0.011
Mechanical	21 (20.0)	6 (11.8)	15 (27.8)	0.052
Neurological	14 (13.5)	2 (3.9)	12 (22.6)	0.008
Pulmonary	8 (7.6)	1 (2.0)	7 (13.0)	0.061
Renal	74 (71.2)	28 (54.9)	46 (86.8)	< 0.001
**Prediction Scores**				
PEP model	45.0 (40.0, 56.0)	42.0 (38.0, 50.0)	51.0 (42.0, 61.5)	0.001
Precannulation Pedi-SAVE	49.0 (46.0, 53.0)	50.0 (47.0, 53.0)	49.0 (45.0, 53.0)	0.127
Postcannulation Pedi-SAVE	97.0 (85.0, 111.5)	108.0 (98.0, 118.0)	89.0 (78.5, 97.0)	< 0.001
**Clinical outcomes**				
ECMO duration (h)	123.0 (90.5, 167.0)	100.0 (89.0, 135.0)	145.5 (99.8, 214.3)	< 0.001
Successful Weaning	72 (68.6)	51 (100.0)	21 (38.9)	< 0.001
Hospital length of stay (d)	41.0 (22.0, 59.0)	51.0 (36.0, 84.0)	24.0 (12.8, 45.0)	< 0.001
ICU length of stay (d)	27.0 (10.5, 46.5)	33.0 (18.0, 56.0)	14.0 (7.0, 35.8)	< 0.001
Ventilation time (h)	400.0 (188.5, 843.5)	557.0 (282.0, 960.0)	239.5 (142.8, 776.0)	0.004

Notes: Continuous data are presented as median (interquartile range) and categorical data as n (percent). ECMO, Extracorporeal Membrane Oxygenation; RACHS-1, Risk Adjustment for Congenital Heart Sugery-1; STAT, Society of Thoracic Surgeons-European Association of Cardiothoracic Surgery; CPB, cardiopulmonary bypass; ECPR, extracorporeal cardiopulmonary resuscitation; MAP, mean arterial pressure; VIS, vasoactive-inotropic score; APTT, activated partial thromboplastin time, INR international normalized ratio; PEP, Pediatric Extracorporeal Membrane Oxygenation Prediction; Pedi-SAVE, Pediatric Survival After Veno-arterial ECMO; ICU, intensive care unit.
